# Identification of the active ingredients and pharmacological effects of Kuntai capsules in the treatment of primary ovarian insufficiency: A review

**DOI:** 10.1097/MD.0000000000033884

**Published:** 2023-05-26

**Authors:** Feng-Xia Liu, Yan Sun

**Affiliations:** a Guangxi Medical University, Nanning, China; b The Reproductive Hospital of Guangxi Zhuang Autonomous Region, Nanning, China.

**Keywords:** Kuntai, molecular docking, network pharmacology, primary ovarian insufficiency

## Abstract

Kuntai capsules are effective in controlling primary ovarian insufficiency (POI). However, the precise mechanisms underlying the pharmacological effects of Kuntai capsules remain unclear. This study aimed to screen the active components and underlying mechanisms of Kuntai capsules for POI treatment using network pharmacology protocols and molecular docking technology. Potential active constituents in the chemical composition of Kuntai capsules were obtained from the Traditional Chinese Medicine System Pharmacology Database. Targets for POI were obtained from the Online Mendelian Inheritance in Man and Gene Cards database. All target data were integrated to identify the active ingredients of POI treatment. Enrichment analyses were performed using the Database for Annotation, Visualization, and Integrated Discovery database. The STRING database and Cytoscape software were used for protein-protein interaction network construction and core target identification. Finally, a molecular docking analysis of the active components and core targets was performed. A total of 157 ingredients related to POI were identified. Enrichment analysis showed that these components might participate in the mitogen-activated protein kinase, tumor necrosis factor, phosphoinositide-3-kinase/AKT serine/threonine kinase 1, and forkhead box O signaling pathways. Further protein-protein interaction network analysis revealed that the core targets were Jun proto-oncogene, AKT serine/threonine kinase 1, tumor protein P53, interleukin 6, and the epidermal growth factor receptor. Molecular docking analysis showed that baicalein was the most active ingredient with the highest affinity for the core targets. This study identified baicalein as the core functional component and elucidated the potential pharmacological effects of Kuntai capsule in the treatment of POI.

## 1. Introduction

Primary ovarian insufficiency (POI) is an ovarian function disorder that occurs before the age of 40 years and causes permanent and irreversible damage to the ovarian function. The diagnostic criteria for POI are oligomenorrhea or amenorrhea at least 4 months before 40 years of age, with serum follicle-stimulating hormone levels > 25 IU/L (on 2 occasions at least 1 month apart).^[1]^ The etiology of POI includes genetic and iatrogenic factors, infections, and autoimmune disorders (ovarian surgery and chemotherapy).^[2–4]^ A meta-analysis revealed that the worldwide incidence of POI in 2019 was 3.7%.^[5]^ Currently, there are no effective therapies for POI.^[6]^ Hormone replacement therapy (HRT) is the first-line therapy to improve low estrogen levels in patients with POI, which may prevent cardiovascular diseases and bone issues.^[7]^ However, the clinical use of HRT is limited by its side effects and contraindications, especially in patients with neoplastic or endocrine complications.^[8,9]^ In addition, HRT has little effect on fertility, although many women with POI still have reproductive needs. Therefore, there is an urgent need to develop more effective replacement drugs for POI with fewer side effects.

Traditional Chinese Medicine (TCM) has been used in combination with HRT to treat POI.^[10,11]^ Of these TCM, the Kuntai capsule, which originated from a modified Huanglian Ajiao decoction published by Shang Han Za Bing Lun, is an efficient intervention for ovarian dysfunction.^[12,13]^ The Kuntai capsule is a compound herbal formula comprising 6 herbs: *Radix Rehmanniae Praeparata* (shudihuang), *Rhizoma Coptidis* (huanglian), *Radix Paeonia Alba* (baishao), *Radix Scutellaria Baicalensis* (huangqin), Poria Cocos (fuling), and donkey-hide gelatin (ejiao). A meta-analysis involving 12 randomized controlled trials revealed that combined treatment with Kuntai capsules and HRT is more effective than HRT alone for POI treatment, including improved serum levels of anti-Mullerian hormone, estradiol, follicle-stimulating hormone, and luteinizing hormone, lipid indexes, and ultrasonic results (antral follicle count, ovarian diameter, ovarian resistance index, peak systolic velocity, and perfusion index).^[14]^ Similarly, a previous study showed that combined therapy with Kuntai capsules and HRT had a higher therapeutic efficacy rate than HRT monotherapy.^[15]^ Moreover, clinical trials have indicated the benefits of Kuntai capsule administration for POI, especially in fertility treatment, including elevation of serum anti-Müllerian hormone levels, estrogen levels, antral follicle counts, increased spontaneous pregnancy rates, and improved assisted reproductive technology outcomes.^[12]^ Luo et al^[16]^ found that Kuntai capsules can improve ovarian function by maintaining follicles and activating autophagy using a cisplatin-induced POI rat model. However, the exact mechanism of Kuntai capsules in the treatment of POI is still unclear. Network pharmacology with molecular docking is a novel approach for revealing the potential mechanisms underlying the therapeutic effects of a drug through integrating drug-target-disease networks with several bioinformatics methods and binding ability verification.^[17,18]^ Therefore, it is appropriate to elucidate the molecular mechanisms of Kuntai capsule in treatment of POI using network pharmacology with molecular docking, instead of conventional analytical strategies because the effects of Kuntai capsules on POI are complex and may result from multiple components acting on multiple targets and multiple pathways. To the best of our knowledge, no such studies have been reported to date.

**Figure 1.**
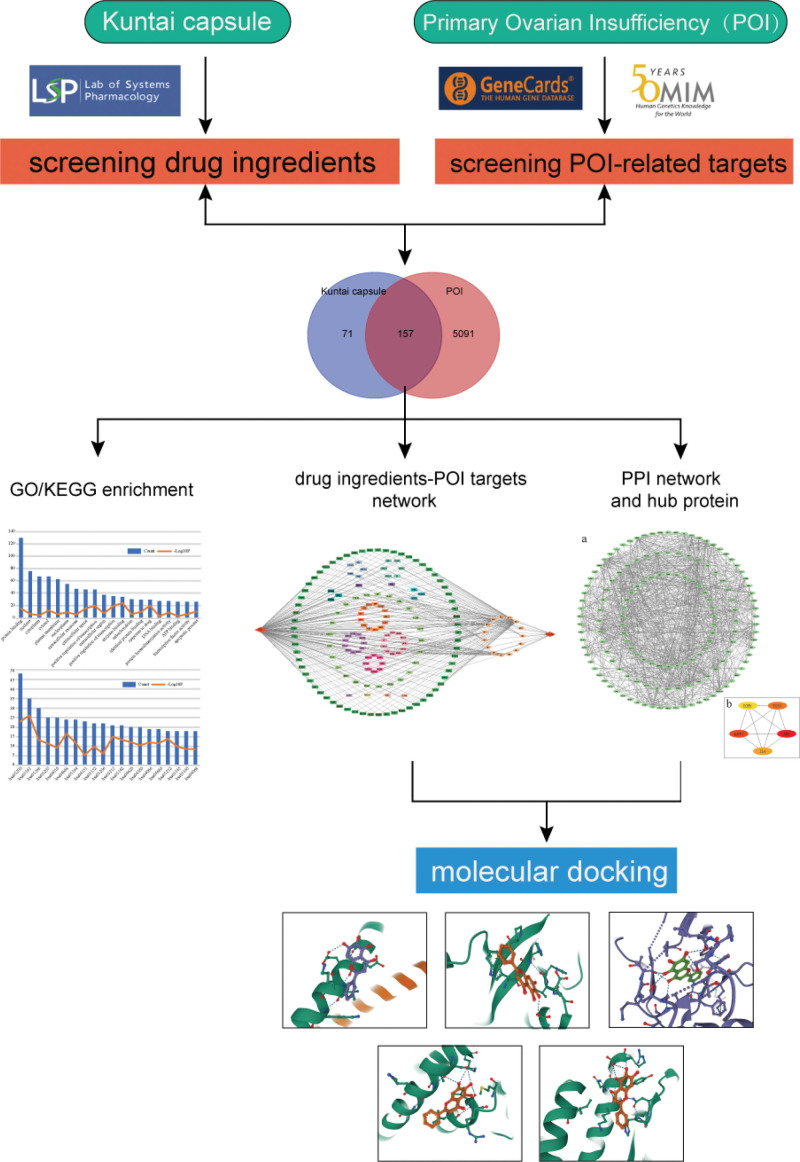
Flow chart of the study.

To explore the potential mechanisms of Kuntai capsules involved in the treatment of POI, we screened the active components of Kuntai capsules as well as potential treatment targets of POI using a network pharmacology approach, and further verified the binding ability between components and targets by running molecular docking. This study revealed the most active component and therapeutic mechanisms of Kuntai capsules against POI, which may provide a new rationale for basic research and lead to new drug development for treating POI. A flow chart of the study is shown in Figure 1.

## 2. Methods

### 2.1. Screening for the main ingredients of Kuntai capsules

The TCM system pharmacology database and analysis platform (TCMSP), (http://tcmspw.com/index.php) is a public pharmacology platform for TCM analysis that includes chemical, target, and drug-target networks.^[19]^ We collected the predicted targets of the active ingredients of Kuntai capsules using TCMSP. The criteria for screening active ingredients included a drug-likeness evaluation equal to or >0.18 and an oral bioavailability equal to or >30%. These 2 parameters are strongly correlated with the absorption, distribution, metabolism, and excretion features of the ingredients. We integrated the data and removed duplicates after obtaining the entire list of active ingredients.

### 2.2. Identification of Kuntai capsule ingredients impacting POI

GeneCards (https://www.genecards.org) is a public database that provides comprehensive information regarding human genes.^[20]^ The OMIM (http://www.omim.org/) is an authoritative resource for all Mendelian disorders and over 16,000 genes associated with them.^[21]^ We obtained POI-related target genes from the Gene Cards and OMIM databases. To identify the candidate targets of Kuntai capsules impacting POI, we combined the predicted targets of the active ingredients of Kuntai capsules and POI-related genes using the Venn diagram online tool (http://bioinformatics.psb.ugent.be/webtools/Venn/). The Cytoscape software platform is an open-source network analysis and visualization tool for biological research.^[22]^ Ingredient-gene networks were obtained and visualized using the Cytoscape software.

### 2.3. Gene ontology/Kyoto encyclopedia of genes and genomes (GO/KEGG) enrichment analysis

GO annotation describes a specific gene from different dimensions, including biological process, molecular function, and cellular component. KEGG pathway enrichment analysis provides a collection of pathway maps that represent the molecular interactions of several genes. GO term and KEGG pathway enrichment analyses were performed for candidate target genes using the Database for Annotation, Visualization, and Integrated Discovery (https://david.ncifcrf.gov/).^[23]^ The GO terms and KEGG pathway term clusters (*P* values < .05) were selected, and the top 20 terms were ranked according to the gene count.

### 2.4. Protein-protein interaction (PPI) network and hub protein identification

STRING (https://www.string-db.org/) is an online resource that integrates protein interaction information with confidence scores. The PPI network was established using the STRING database (version 11.5) (https://string-db.org/) and visualized using the Cytoscape software (version 3.8.2) (https://cytoscape.org/index.html). In the PPI network, 0.700 (high-confidence) interaction nodes were required, and the disconnected nodes were hidden. The PPI network was visualized using the Cytoscape software, and further analysis was performed using the Cytoscape plugin. CytoHubba is a plugin that explores important nodes (also known as hub proteins) of a network using several algorithms. Hub proteins were identified based on the PPI network using CytoHubba version 0.1 (https://apps.cytoscape.org/apps/cytohubba) and Maximal Clique Centrality algorithms.

### 2.5. Molecular docking

Molecular docking is a method for identifying and visualizing the interactions between receptors and ligands. The Protein Database (PDB) (http://www.rcsb.org/) was used to download the 3D structures of hub proteins. The molecular structures of quercetin, kaempferol, and baicalein were downloaded from PubChem (https://pubchem.ncbi.nlm.nih.gov/).^[24]^ Water molecules were removed from all protein and ingredient molecules, polar hydrogen atoms were added, and the samples were stored in PDBQT format. The AutodockVina 1.2.2 (http://autodock.scripps.edu) was used to analyze the binding affinities between the active ingredients and hub target proteins.^[25]^ The grid box parameters were as follows: box size, 30 × 30 × 30 Å; grid point distance, 0.05 nm.

## 3. Results and discussion

TCM has been used for thousands of years to treat and prevent illnesses.^[26,27]^ However, the development of TCM, which is experience-based rather than evidence-based, is limited by several factors. It is difficult to screen the exact therapeutic targets of each herbal medicine in compound preparations using conventional experimental methods.^[28]^ The central concept of network pharmacology is multi-drug, multi-target, and multi-pathway, which corresponds to the characteristics of TCM preparations.^[29]^ Therefore, network pharmacology, an efficient approach for clarifying ingredient targets and molecular mechanisms, provides new insights into the mechanisms and targets of TCM.^[30]^ This study primarily aimed to identify the active ingredients and pharmacological effects of Kuntai capsules for the treatment of POI using network pharmacology and molecular docking. We identified baicalein as the core active ingredient of Kuntai capsules against POI and identified Jun proto-oncogene (JUN), AKT serine/threonine kinase 1 (AKT1), tumor protein P53 (TP53), interleukin 6 (IL-6), and epidermal growth factor receptor (EGFR) as the key treatment targets for POI.

### 3.1. Ingredient-target network analysis

**Figure 2.**
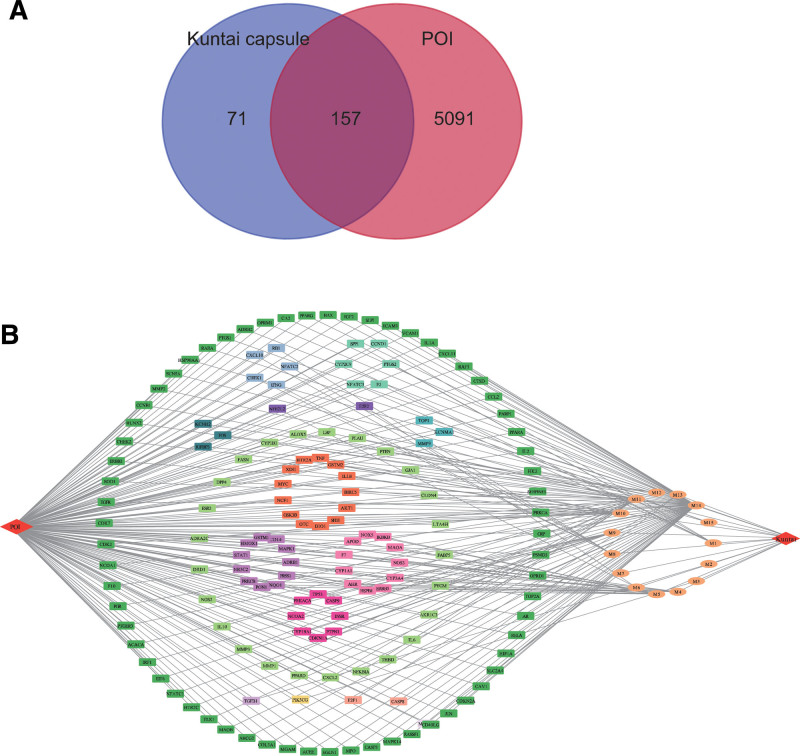
(A) Venn diagrams showing the common molecules of Kuntai capsule ingredients and POI-related targets. (B) The drug ingredients-POI targets network. The square nodes represent POI-related targets; lines represent interaction relationships; and ellipse nodes represent Kuntai capsule ingredients. M1= (+)-catechin; M2 = (2R)-2-[(3S,5R,10S,13R,14R,16R,17R)-3,16-dihydroxy-4,4,10,13,14-pentamethyl-2,3,5,6,12,15,16,17-octahydro-1H-cyclopenta[a]phenanthren-17-yl]-6-methylhept-5-enoic acid; M3 = (3S,5R,8R,9R,10S,14S)-3,17-dihydroxy-4,4,8,10,14-pentamethyl-2,3,5,6,7,9-hexahydro-1H-cyclopenta[a]phenanthrene-15,16-dione; M4 = (R)-canadine; M5 = 5,2’-dihydroxy-6,7,8-trimethoxyflavone; M6 = 5,7,2,5-tetrahydroxy-8,6-dimethoxyflavone; M7 = 5,7,4’-trihydroxy-6-methoxyflavanone, M8 = 5,7,4’-trihydroxy-8-methoxyflavone, M9 = acacetin, M10 = baicalein, M11 = kaempferol, M12 = oroxylin a, M13 = paeoniflorin, M14 = quercetin, M15 = stigmasterol, POI = primary ovarian insufficiency.

A total of 228 ingredients from each herb (except ejiao) of the Kuntai capsules were screened using TCMSP. To identify the Kuntai capsule ingredients involved in POI treatment, we obtained 5248 POI-related genes from GeneCards and OMIM and integrated them with 228 ingredients from TCMSP. Accordingly, 157 common molecules which may involve in the therapeutic effects of Kuntai capsules in treatment of POI were selected (Fig. 2A).

The common molecules included 15 active ingredients, including: (+)-catechin; (2R)-2-[(3S,5R,10S,13R,14R,16R,17R)-3,16-dihydroxy-4,4,10,13,14-pentamethyl-2,3,5,6,12,15,16,17-octahydro-1H-cyclopenta[a]phenanthren-17-yl]-6-methylhept-5-enoic acid; (R)-canadine; 5,7,4’-trihydroxy-6-methoxyflavanone; 5,2’-dihydroxy-6,7,8-trimethoxyflavone; (3S,5R,8R,9R,10S,14S)-3,17-dihydroxy-4,4,8,10,14-pentamethyl-2,3,5,6,7,9-hexahydro-1H-cyclopenta[a]phenanthrene-15,16-dione; 5,7,2,5-tetrahydroxy-8,6-dimethoxyflavone; 5,7,4’-trihydroxy-8-methoxyflavone; acacetin; baicalein; kaempferol; oroxylin a; paeoniflorin; quercetin; and stigmasterol. We constructed a network of drug ingredients-POI targets using the Cytoscape software, as shown in Figure 2B.

The top 3 ingredients, which were the main components with the highest target connection, were identified in the drug ingredients-POI targets network as quercetin, kaempferol, and baicalein. Quercetin is a well-known antioxidant that can prevent primordial follicle loss in mice with iatrogenic POI induced by cyclophosphamide treatment through the phosphoinositide-3-kinase (PI3K)/AKT pathway.^[31]^ Quercetin may also protect ovarian function by decreasing oxidative activity.^[32,33]^ Kaempferol is a natural flavonoid from plants that is involved in several bioprocesses and has antioxidant, anti-inflammatory, and anticancer activities.^[34]^ Kaempferol can promote sheep primordial follicle activation and induces sheep-growing follicle development via the PI3K/AKT pathway.^[35]^ Baicalein is a flavonoid compound mainly found in the roots of huangqin (also known as *Scutellaria baicalensis*). It has been used to treat various diseases owing to its various pharmacological effects, such as antioxidant, anticancer, and anti-inflammatory effects.^[36]^ Baicalein has progesterone receptor antagonist and glucocorticoid receptor agonist activity in vitro, which may have a potent therapeutic effect on female reproductive system disease.^[37]^ Notably, these 3 components share the same pharmacological antioxidant activity. Moreover, it is increasingly believed that oxidative stress may contribute to POI pathogenesis.^[38]^ Patients with POI have increased plasma levels of advanced oxidation protein products, which induce reactive oxygen species generation, resulting in the inhibition of ovarian granulosa cell proliferation, which could lead to the occurrence of POI in vivo.^[39]^ Some antioxidants, such as melatonin, resveratrol, and dehydroepiandrosterone have considerable beneficial effects on the ovarian reserve by reducing oxidative stress.^[40–42]^ Therefore, these results suggest that quercetin, kaempferol, and baicalein in Kuntai capsules may play important roles in the treatment of POI.

### 3.2. GO/KEGG enrichment analysis

**Figure 3.**
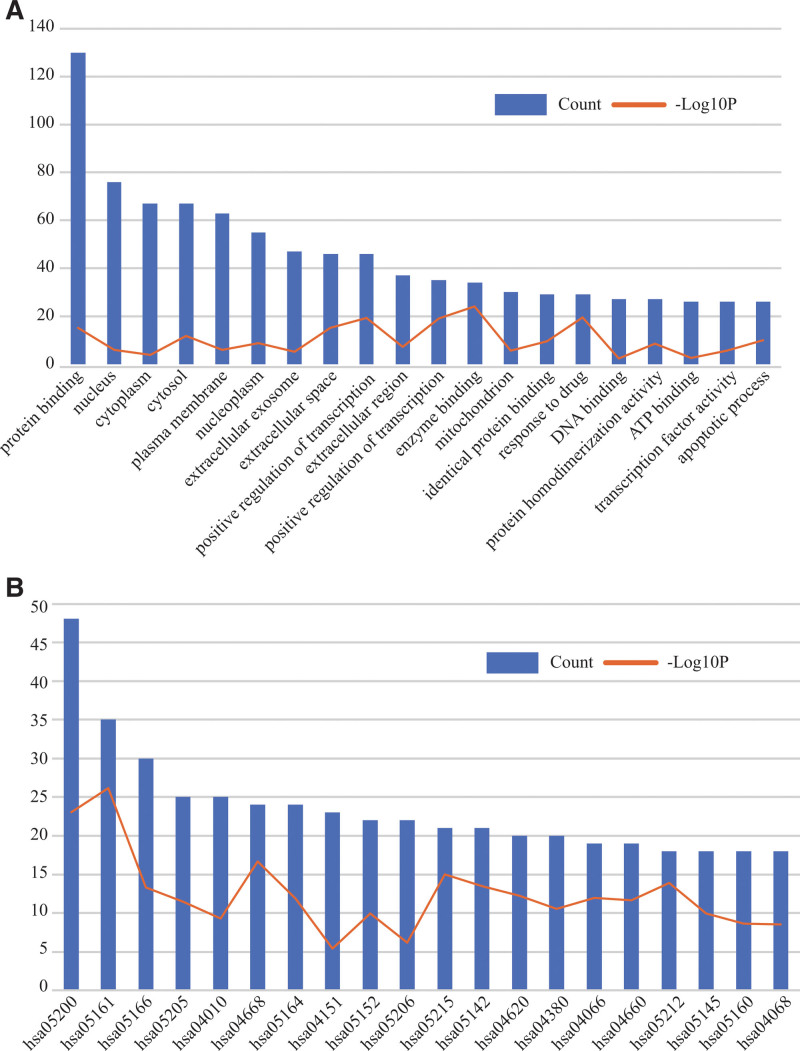
Enrichment analysis. (A) Analysis of the top 20 GO terms (*P* < .05), (B) analysis of the top 20 KEGG pathways (*P* < .05). GO = gene ontology, KEGG = Kyoto encyclopedia of genes and genomes.

GO and KEGG enrichment analyses of the candidate target genes were performed using database for annotation, visualization, and integrated discovery. The top 20 terms are listed in Table 1 and Table 2 and in Figure 3A and B (*P* < .05; arranged by gene count). According to the GO enrichment analysis, the candidate target genes were mainly involved in protein binding, DNA binding, ATP binding, positive regulation of transcription from the RNA polymerase II promoter, protein homodimerization activity, and apoptosis. KEGG pathway analysis revealed that the candidate genes were related to the mitogen-activated protein kinase (MAPK), tumor necrosis factor, PI3K/AKT, and forkhead box O (FOXO) signaling pathways.

**Table 1:** The top 20 terms of GO enrichment analysis.

Category	Terms	Count
MF	Protein binding	130
CC	Nucleus	76
CC	Cytoplasm	67
CC	Cytosol	67
CC	Plasma membrane	63
CC	Nucleoplasm	55
CC	Extracellular exosome	47
CC	Extracellular space	46
BP	Positive regulation of transcription from RNA polymerase II promoter	46
CC	Extracellular region	37
BP	Positive regulation of transcription, DNA-templated	35
MF	Enzyme binding	34
CC	Mitochondrion	30
MF	Identical protein binding	29
BP	Response to drug	29
MF	DNA binding	27
MF	Protein homodimerization activity	27
MF	ATP binding	26
MF	Transcription factor activity, sequence-specific DNA binding	26
BP	Apoptotic process	26

*P* < .05.

BP = Biological Process, CC = Cellular Component, GO = gene ontology, MF = Molecular Function.

**Table 2:** The top 20 terms of KEGG pathway enrichment analysis.

Category	Terms	Count
hsa05200	Pathways in cancer	48
hsa05161	Hepatitis B	35
hsa05166	HTLV-I infection	30
hsa05205	Proteoglycans in cancer	25
hsa04010	MAPK signaling pathway	25
hsa04668	TNF signaling pathway	24
hsa05164	Influenza A	24
hsa04151	PI3K-Akt signaling pathway	23
hsa05152	Tuberculosis	22
hsa05206	MicroRNAs in cancer	22
hsa05215	Prostate cancer	21
hsa05142	Chagas disease (American trypanosomiasis)	21
hsa04620	Toll-like receptor signaling pathway	20
hsa04380	Osteoclast differentiation	20
hsa04066	HIF-1 signaling pathway	19
hsa04660	T cell receptor signaling pathway	19
hsa05212	Pancreatic cancer	18
hsa05145	Toxoplasmosis	18
hsa05160	Hepatitis C	18
hsa04068	Foxo signaling pathway	18

*P* < .05.

KEGG = Kyoto encyclopedia of genes and genomes, MAPK = mitogen-activated protein kinase, PI3K = phosphoinositide-3-kinase, TNF = tumor necrosis factor.

GO and KEGG enrichment results indicated biological characteristics of the candidate target genes, from which we can learn the underlying pharmacological mechanism of Kuntai capsules in the treatment of POI. GO enrichment analysis revealed that the targets were mainly enriched for biological processes such as transcription, which suggests that the pharmacological effects of Kuntai capsules on POI may involve in the transcription regulation of POI target genes. Inappropriate follicular development, especially the depletion of primordial follicles, is a prominent event in the progression of POI.^[43]^ The MAPK, PI3K-AKT, and FOXO signaling pathways, which were enriched in KEGG enrichment analyses, are classic pathways in the regulation of follicular development and are also involved in POI.^[44,45]^ A previous study suggested that quercetin prevents primordial follicle loss in mice with cyclophosphamide-induced POI by inhibiting the PI3K-AKT-FOXO3a pathway.^[31]^ The MAPK pathway is a downstream pathway that is related to oxidative stress. MAPK and PI3K-AKT pathways play important roles in follicle activation and POI.^[46]^ Moreover, the tumor necrosis factor pathway plays an essential role in inflammation and can induce POI via apoptosis.^[47]^

### 3.3. Hub protein identification and molecular docking

**Figure 4.**
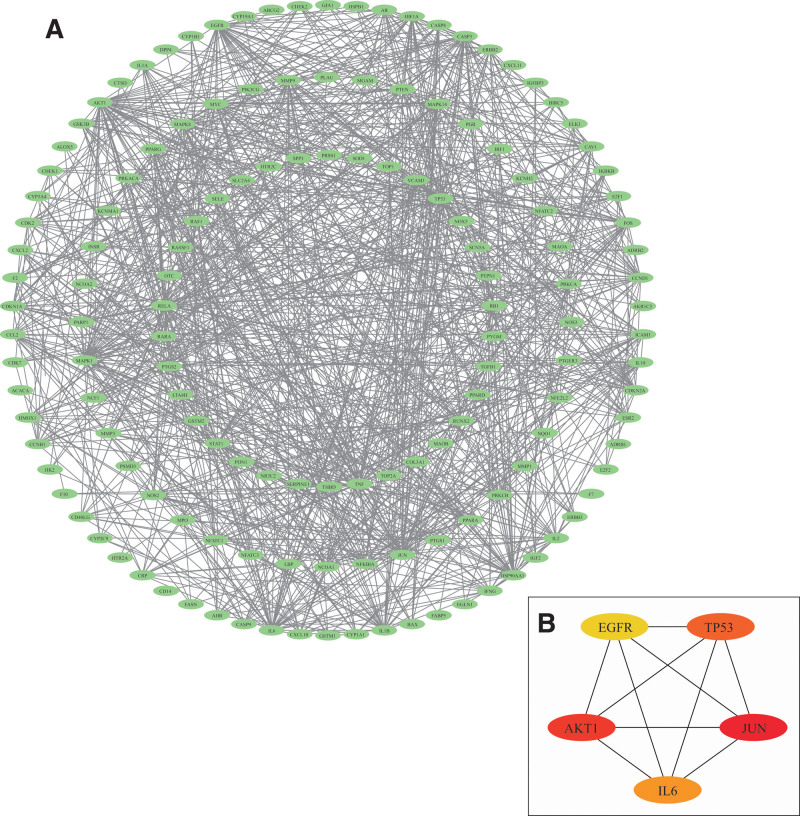
Network visualization. (A) PPI network of 157 intersected targets with an interaction score ≥ 0.7. (B) Hub proteins of the PPI network identified by the MMC method. The darker the color, the higher the degree of the node. MMC = maximal clique centrality, PPI = protein-protein interaction.

The PPI network of 157 intersecting molecules was obtained using STRING software. Visualization of the PPI network is shown in Figure 4A. In the PPI network of 144 nodes and 985 edges were identified in the PPI network. To further analyze the PPI network, we selected the top 5 hub proteins (JUN, AKT1, TP53, IL-6, and EGFR) using CytoHubba (a plugin of Cytoscape software), as shown in Figure 4B. Hub proteins were highly connected in the network, suggesting that they may be significant therapeutic targets that require further verification. Genes are colored according to maximal clique centrality algorithm scores. The details of the top 10 hub proteins are listed in Table 3.

**Table 3:** Top 5 genes in the PPI network ranked by the MCC method.

Rank	Name	Description	Score
1	JUN	Jun proto-oncogene, AP-1 transcription factor subunit	2963178.0
2	AKT1	AKT serine/threonine kinase 1	2833772.0
3	TP53	Tumour protein P53	2610781.0
4	IL-6	Interleukin 6	2050512.0
5	EGFR	Epidermal growth factor receptor	1759416.0

AKT1 = AKT serine/threonine kinase 1, EGFR = epidermal growth factor receptor, IL-6 = interleukin 6, JUN = Jun proto-oncogene, MCC = Maximal Clique Centrality, PPI = protein-protein interaction, TP53 = tumor protein P53.

**Figure 5.**
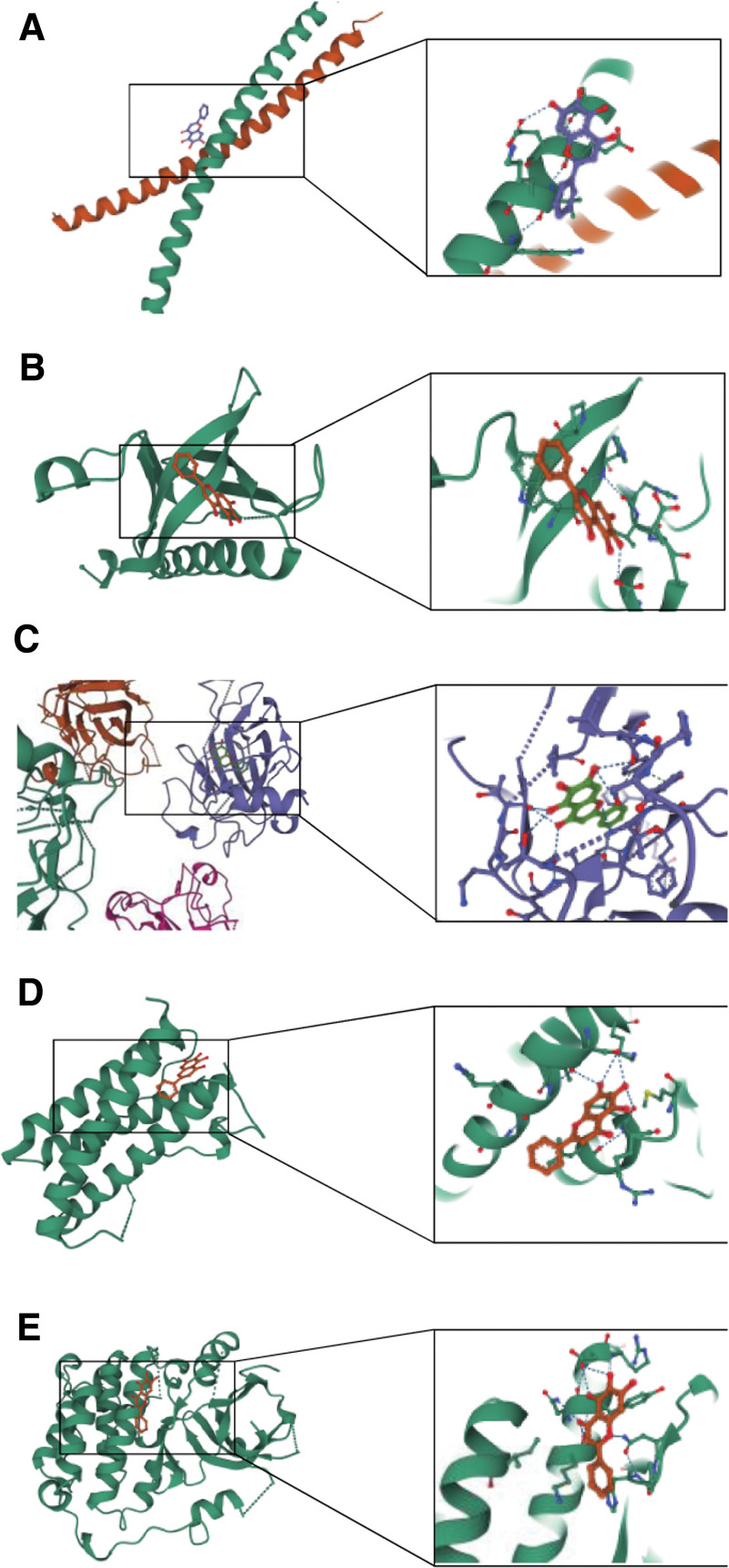
Molecular docking of baicalein with hub proteins. (A) Baicalein-JUN (binding energy: −5.520). (B) Baicalein-AKT1(binding energy: −5.784). (C) Baicalein-TP53 (binding energy: −7.860). (D) Baicalein-IL-6 (binding energy: −6.323). (E) Baicalein-EGFR (binding energy: −5.240). AKT1 = AKT serine/threonine kinase 1, EGFR = epidermal growth factor receptor, IL-6 = interleukin 6, JUN = Jun proto-oncogene, TP53 = tumor protein P53.

To further explore the interaction between core active ingredients and hub proteins, we selected the top 3 ingredients (quercetin, kaempferol, and baicalein) based on the drug ingredients-POI targets network and PPI results to conduct molecular docking with hub proteins: JUN (PDB ID, 1JNM), AKT1 (PDB ID, 1H10), TP53 (PDB ID, 7B46), IL-6 (PDB ID, 1LAU), and EGER (PDB ID, 3POZ). The interactions between the 3 ingredients and 5 proteins were analyzed using AutoDock Vina version 1.2.2. Binding energy is a common index used to evaluate the docking between receptors and ligands. Binding energies below − 5 kcal/mol indicate a good connection between the receptor and ligand.^[48,49]^ The binding energies for each docking molecule are presented in Table 4. According to the results, quercetin, kaempferol, and baicalein could molecularly dock with hub proteins to varying degrees depending on the binding energy. However, the combination of baicalein and core proteins (JUN, AKT1, TP53, IL-6, and EGFR) was more stable and had a higher affinity with the lowest binding energy, indicating that baicalein may be the main active component of the Kuntai capsule in the treatment of POI. The interaction fractions between baicalein and the hub proteins are shown in Figure 5. At present, there are no in vivo or in vitro studies in the literature to verify the treatment mechanism of baicalein in POI. Our study suggests that investigating the regulatory roles of the targets and signaling pathways of the active ingredients of the Kuntai capsule, particularly baicalein, for treating POI may be a crucial step toward a better understanding of the condition. According to our findings, elucidating the exact mechanism of Kuntai capsule on POI continues to be a challenge; therefore further, more comprehensive experiments are required.

**Table 4:** The affinity of ingredients and hub proteins.

Ingredient	Binding energy (kcal/mol)				
	JUN	AKT1	TP53	IL-6	EGER
Quercetin	−5.213	−5.087	−3.058	−6.110	−4.990
Kaempferol	−5.372	−5.367	−5.94	−5.928	−4.484
Baicalein	−5.520	−5.784	−7.860	−6.323	−5.240

AKT1 = AKT serine/threonine kinase 1, EGFR = epidermal growth factor receptor, IL-6 = interleukin 6, JUN = Jun proto-oncogene, TP53 = tumor protein P53.

## 4. Conclusions

In this study, we explored the mechanisms by which Kuntai capsules exert therapeutic pharmacological effects on POI and identified potential therapeutic targets and pathways using network pharmacology. In addition, by molecular docking, we identified baicalein as the core component of the Kuntai capsule involved in the treatment of POI. These findings extend understanding of the mechanisms of Kuntai capsules for treating POI, while also providing scope for additional research for new drug development.

## Acknowledgments

We acknowledge the public databases for providing their platforms and contributors for uploading meaningful data. We would like to thank Editage (www.editage.cn) for the English language editing.

## Author contributions

**Funding acquisition:** Yan Sun.

**Supervision:** Yan Sun.

**Writing – original draft:** Feng-Xia Liu.
